# *My 28 Days* - a global digital women’s health initiative for evaluation and management of secondary amenorrhea: case report and literature review

**DOI:** 10.3389/fendo.2023.1227253

**Published:** 2023-09-12

**Authors:** Lawrence M. Nelson, Hillary Spencer, Karima Hijane, Payom Thinuan, Chaninan W. Nelson, Amanda J. Vincent, Catherine M. Gordon, Tony M. Plant, Pouneh K. Fazeli

**Affiliations:** ^1^ Digital Women's Health Initiative, Mary Elizabeth Conover Foundation, Tysons, VA, United States; ^2^ Faculty of Nursing, Boromarajonani College of Nursing Nakhon, Lampang, Thailand; ^3^ Monash Centre for Health Research and Implementation (MCHRI), Monash University, Clayton, VIC, Australia; ^4^ Endocrinology and Metabolism, Baylor College of Medicine, Houston, TX, United States; ^5^ Endocrinology and Metabolism, University of Pittsburgh School of Medicine, Pittsburgh, PA, United States

**Keywords:** amenorrhea - diagnosis, therapy, digital medical, policy & institutional actions, preventative medicine, physiology reproduction, estradiol (17ß-estradiol), hormone replacement therapy

## Abstract

There is a need to close the gap between knowledge and action in health care. Effective care requires a convenient and reliable distribution process. As global internet and mobile communication increase capacity, innovative approaches to digital health education platforms and care delivery are feasible. We report the case of a young African woman who developed acute secondary amenorrhea at age 18. Subsequently, she experienced a 10-year delay in the diagnosis of the underlying cause. A global digital medical hub focused on women’s health and secondary amenorrhea could reduce the chance of such mismanagement. Such a hub would establish more efficient information integration and exchange processes to better serve patients, family caregivers, health care providers, and investigators. Here, we show proof of concept for a global digital medical hub for women’s health. First, we describe the physiological control systems that govern the normal menstrual cycle, and review the pathophysiology and management of secondary amenorrhea. The symptom may lead to broad and profound health implications for the patient and extended family members. In specific situations, there may be significant morbidity related to estradiol deficiency: (1) reduced bone mineral density, 2) cardiovascular disease, and 3) cognitive decline. Using primary ovarian insufficiency (POI) as the paradigm condition, the Mary Elizabeth Conover Foundation has been able to address the specific global educational needs of these women. The Foundation did this by creating a professionally managed Facebook group specifically for these women. POI most commonly presents with secondary amenorrhea. Here we demonstrate the feasibility of conducting a natural history study on secondary amenorrhea with international reach to be coordinated by a global digital medical hub. Such an approach takes full advantage of internet and mobile device communication systems. We refer to this global digital women’s health initiative as *My 28 Days^®^*.

## Case report

A 28-year-old nulligravid woman from Africa who had never given birth to a child (nulligravid) experienced the sudden cessation of menstrual periods at age 18 (amenorrhea). She gave informed consent to publish her experience as a case report with no personal identification data included.

She had breast development (thelarche) at age 9, first menses (menarche) at age 11, and subsequently established regular monthly menstrual periods lasting 5 days. She delayed seeing a clinician about the amenorrhea for 10 months. She had assumed the amenorrhea was stress related. She experienced mild “hot flashes” (vasomotor symptoms) with the onset of amenorrhea. Over the next 3 years the vasomotor symptoms intensified and she developed night sweats, fatigue, and “brain fog”. At age 19 she saw a general practitioner who performed unspecified laboratory tests and told her she had “low estrogen”. The practitioner referred her to a specialist who had a long wait list. She did not pursue the situation further at that time.

At age 21 the amenorrhea persisted and her vasomotor symptoms became more intense. She consulted a gynecologist who performed no laboratory tests and prescribed a progestin challenge test to “kick start” her period. She had a withdrawal bleed in response to the progestin challenge. The amenorrhea and vasomotor symptoms continued unabated and the woman was frustrated by these events. She did not return to the gynecologist until one year later, at which time the gynecologist repeated the progestin challenge test and told her that if her periods did not return after this intervention there was nothing more to do. The woman again experienced bleeding in response to the progestin challenge, but the amenorrhea and intense vasomotor symptoms continued unabated. Based on the clinician’s statements, the woman concluded she had idiopathic amenorrhea of unknown cause (idiopathic) and that nothing more could be done for her. In 2017 at age 24 she developed idiopathic urticaria and angioedema which spontaneously resolved over the ensuing year. She has no family history of POI.

In February of 2021, at the age of 28, six years after her last gynecologic consultation, and fully ten years after the onset of amenorrhea, the woman saw another gynecologist and was finally given a diagnosis of Primary Ovarian Insufficiency (POI). Her laboratory testing then revealed FSH of 50.75 mIU/mL (normal range 2.9-14.6), LH 22.34 mIU/mL (normal range 1.9-14.6), and estradiol <9 pg/mL (normal range 15-350). The clinician performed no tests to determine the potential cause of the POI and did not evaluate her bone mineral density (BMD) or her serum vitamin D level. The woman had no other chronic health conditions. 

After receiving her POI diagnosis in February 2021, the woman searched on Facebook for groups dedicated to POI. She desired to learn more about the condition, its treatment, and to find a community of women who understood the associated life challenges. She joined the two Facebook groups with the largest memberships. She reports, “I soon noticed that many women were referencing something called ‘P-HRT’ [Physiologic HRT] and asked one of them what that was.”. As a result, the woman learned about and joined a Facebook group supported by the Mary Elizabeth Conover Foundation, “A Community Resource for Primary Ovarian Insufficiency” (1).

The woman’s primary motivation for joining the Conover Foundation Facebook group was to learn “how to address my hot flashes because they were raging and debilitating at that point.”. In her words, “And although the gynecologist who diagnosed me was able to identify, based on my hormone panel results, that I had POI, he was clueless about its treatment and only prescribed DHEA supplements along with some other multivitamins called Ovacare which are usually taken by women trying to conceive. It was not until a couple of weeks later when I returned to him to complain about my hot flashes continuing to bother me that he prescribed an oral contraceptive called Microgynon 30.”.

Moreover, she reports “I discovered that I was on the wrong treatment path. By the time I discovered the [Facebook] page, I was taking the Microgynon pills and was under the impression that birth control was also an adequate form of ‘hormone replacement’ since it provides both estrogen and progesterone [progestin]. Your Facebook page helped me learn that P-HRT is the correct treatment for POI because it mimics natural ovarian function, which is the optimal way of replacing the hormones our ovaries no longer make in sufficient amounts. After learning this, I tried to speak to my gynecologist about putting me on P-HRT but he was adamant that birth control is also a form of hormone replacement. By this point I was older and wiser so I knew not to listen to a single doctor’s word as law and decided to seek out another gynecologist who would prescribe P-HRT.”.

In July 2021 the woman contacted the Mary Elizabeth Conover Foundation. Their POI team assessed her situation from a health coaching perspective. This perspective centers on empowering the individual to take control of their health and staying well, rather than simply treating symptoms or conditions. Their process collates the clinical evidence from the patient, educates the patient about POI as a condition, and provides the patient with a coaching referral letter to be taken to a healthcare provider. The woman reported “To his credit though, unlike the gynecologist who diagnosed me, my current doctor is humble, open to learning, and tries to keep up with the latest evidence. So once I shared with him the letter … and the accompanying POI studies, he became convinced of the validity of the evidence and agreed to finally prescribe the exact NIH P-HRT regimen. He also learned a lot about the bone mineral density issue for women with POI from one of the studies … so he now wants me to do a DXA scan to establish a baseline which can be used to evaluate progress.”.

This case emphasizes the need for more aggressive evaluation of secondary amenorrhea (2). Digital healthcare provides a more convenient and effective solution for providing integrated care based on the best evidence (3). The COVID-19 pandemic has driven the rapid expansion of telemedicine use for urgent care and nonurgent care visits including menopause and POI (4–6). In the USA, stakeholders continue to push Congress to expand telehealth care options permanently (7). Telemedicine presents a potential economic boom in developing nations (8).

## Background

There have been calls to establish a global strategy for women’s health care and research (9). A global strategy must represent women’s health concerns more effectively in clinical research, basic science research, translational research, and related leadership positions (10, 11). There is also a need to close the gap between published clinical research and the translation of established findings into clinical practice (12, 13). This will require a frameshift to stimulate more applied research that will identify methods to improve results of care and teach clinicians and researchers how to implement such practices (14). The Institute of Medicine (US) Committee on Quality of Health Care in America has called for change to close the quality gap in care (15). Effective care requires a robust logistical distribution system with supporting policies and infrastructure (16–18).

Recently, in response to a congressional request associated with women’s health research, the US National Institutes of Health hosted an event entitled *“Advancing NIH Research on the Health of Women: A 2021 Conference.” *(19) The key topics discussed, as identified by Congress, were:

1. Rising maternal morbidity and mortality rates.

2. Rising rates of chronic debilitating conditions in women.

3. Plateau in cervical cancer survival rates.

Health care is now much more than local care. Medical tourism is growing actively as global value chains foster international trade in healthcare services (20). International care is made possible by technological advancements in cross-border communication and transportation. The high cost of healthcare services in many nations has created barriers to care for many women. Also, legal constraints have also made specific medical procedures unavailable in some countries (21). Over 70% of adults access the internet to research a health issue (22). However, systematic evaluation of consumer websites has demonstrated significant deficiencies in quality and information content (23), highlighting the need for evidence based digital resources. High quality patient centered health care includes the provision of reliable and accurate information with impacts on patient experience, health literacy, behavior change and outcomes (24, 25).

Women’s reproductive health care is one specific area of concern. Lack of understanding of basic menstrual physiology hampers progress (10, 26). Published evidence demonstrates that women who present with secondary amenorrhea are not promptly evaluated and managed. In one research study, over half of the women who presented with a complaint of amenorrhea reported visiting a clinician’s office three or more times before obtaining proper laboratory testing to determine the diagnosis (2). Most had to see three or more clinicians before a definitive diagnosis was made. In 25% of young women, it took longer than five years to diagnose a rare cause of secondary amenorrhea known as primary ovarian insufficiency (POI) (2).

In 2008 the Developmental Endocrinology Branch of the National Institutes of Health Intramural Research Program (DEB-NIH-IRP) began organizing scientific conferences to bring attention to the menstrual cycle as a vital sign regarding general health. The specific purpose was to raise awareness and advance research on POI (27). The call was to develop an international collaborative approach to menstrual health that “combines the principles of integrative care and community-based participatory research” (28, 29).

Here, the Mary Elizabeth Conover Foundation provides proof of concept for establishing a global digital medical hub for secondary amenorrhea. This private foundation, whose mission is to improve access to health care and education and founded by the first author (LMN), links this effort with an educational initiative called My 28 Days® (https://my28days.org/) (30). The purpose, established in 2017, is to educate patients free of charge so they can be better prepared to take their situation to a clinician for evaluation and management, At the time of submission over 3,000 members had participated in the program. Herein we present proof of concept for a global, seamlessly integrated, mobile health system addressing the care needs of women with POI. As a rare disorder, POI highlights the special challenges facing all people dealing with a rare condition.

This My 28 Days® global approach to a woman's health care 1) links patients and community health providers in real-time, and 2) provides a team having the requisite knowledge and expertise to educate, advocate, and coordinate case management (30). In addition, by providing women with a forum to describe their experiences, benefits may be gained in regard to understanding, emotional support and behavior change. There is a gap between knowledge and action for women presenting with secondary amenorrhea (29, 31). Effective clinicians apply medical evidence in a context relative to the specific needs of the patient. Evidence-based medicine can also be patient-centered medicine (32). In the case presented herein, a clinician told a young woman who presented with secondary amenorrhea that there was nothing more to do after merely administering a progestin challenge test. This delay in diagnosis and management is not acceptable.

An internationally coordinated care and research effort on menstrual cycle health would advance the field more rapidly. The effort must be self-sustaining rather than dependent on philanthropic grants or government support. A digital medical women’s health hub would synergize with medical tourism as global value chains in healthcare services grow (20). Notably, in 2021 Yeganeh et al. justified the creation of an evidence based digital resource for women with early menopause and their healthcare providers as acceptable and useful (33). Further, research assessment showed the early menopause digital resource improved women’s health-related empowerment, illness perception, menopause symptoms, risk perception and knowledge (33).

This report will focus on the evaluation and management of secondary amenorrhea. We will also review the advantages of employing a digital strategy to improve access to menstrual cycle education, integrated care, clinical research, translational research and as a means to gain deeper insights into the relevant basic science. First, however, we describe the normal menstrual cycle and its value as a vital sign of general health.

## The normal menstrual cycle

**Figure 1:**
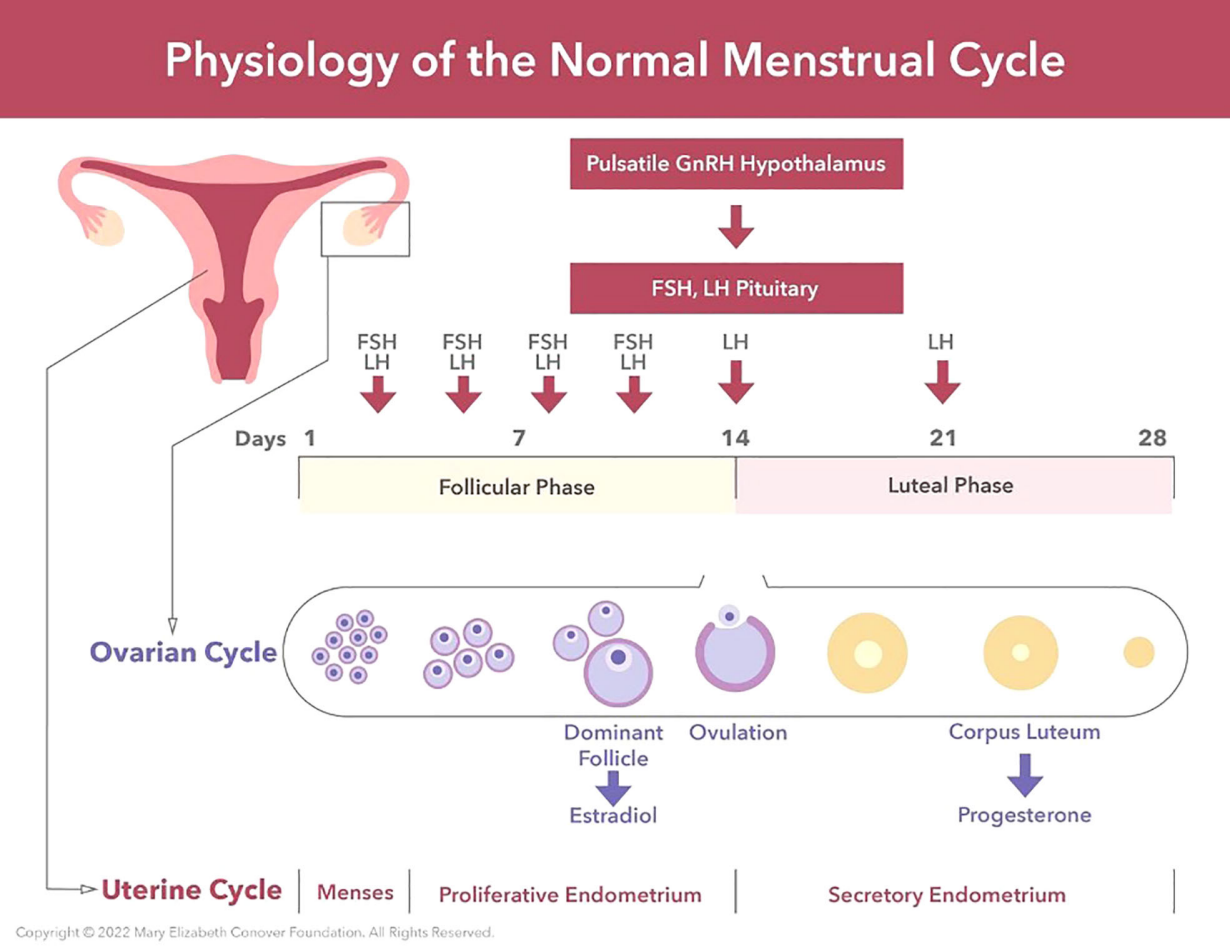
The physiology of the normal menstrual cycle consists of an ovarian cycle and a uterine cycle. The ovarian cycle consists of 1) the follicular phase, and 2) the luteal phase. The follicular phase of the ovarian cycle involves the activation of primordial follicles and the growth of a pool of antral follicles. Follicle growth takes place under the stimulation of FSH and LH from the anterior pituitary. Via a selection process one antral follicle from the pool becomes dominant (the Graafian follicle), secretes large amounts of estradiol for 3 or 4 days which induces an LH surge, and the LH surge triggers ovulation. After ovulation the empty Graafian follicle is transformed into a corpus luteum under the influence of LH secreted by the anterior pituitary. The primary function of the corpus luteum is to secrete progesterone under the stimulation of LH. The uterine cycle comprises 1) menstrual endometrium, 2) proliferative endometrium under the stimulation of serum estradiol, and 3) secretory endometrium under the transforming influence of progesterone. The corpus luteum is programmed to function for 14 days unless rescued by human chorionic gonadotropin (HCG) secreted by an embryo. The menses is induced by falling serum progesterone levels due to waning function of the corpus luteum.

The first day of menstruation is defined as Day 1 of the menstrual cycle. The interval between successive menstruations, and therefore the cycle length, typically ranges from 21-35 days (**Figure 1**). During the first half of the menstrual cycle, termed the follicular phase, the major steroid secreted by the ovary is estradiol and this hormone results in growth and thickening of the inner layer of the uterus known as the endometrium. During the second half of the cycle, the luteal phase, progesterone is also secreted by the ovary and together with estradiol prepares the uterus for the implantation of a blastocyst (multi cell ball-like structure derived from a fertilized egg) should it enter the uterus. Preparation of the uterus for pregnancy involves a thickening of the endometrium with a marked increase in its blood supply. If pregnancy does not occur, the luteal phase terminates after approximately 2 weeks, progesterone and estradiol secretion fall to low levels and stimulation of the uterus by these steroid hormones is withdrawn resulting in a breakdown of the endometrium. The resulting transient uterine bleeding, together with cellular debris shed from degenerating endometrial tissue, comprises the menstrual flow (34–36).

**Figure 2:**
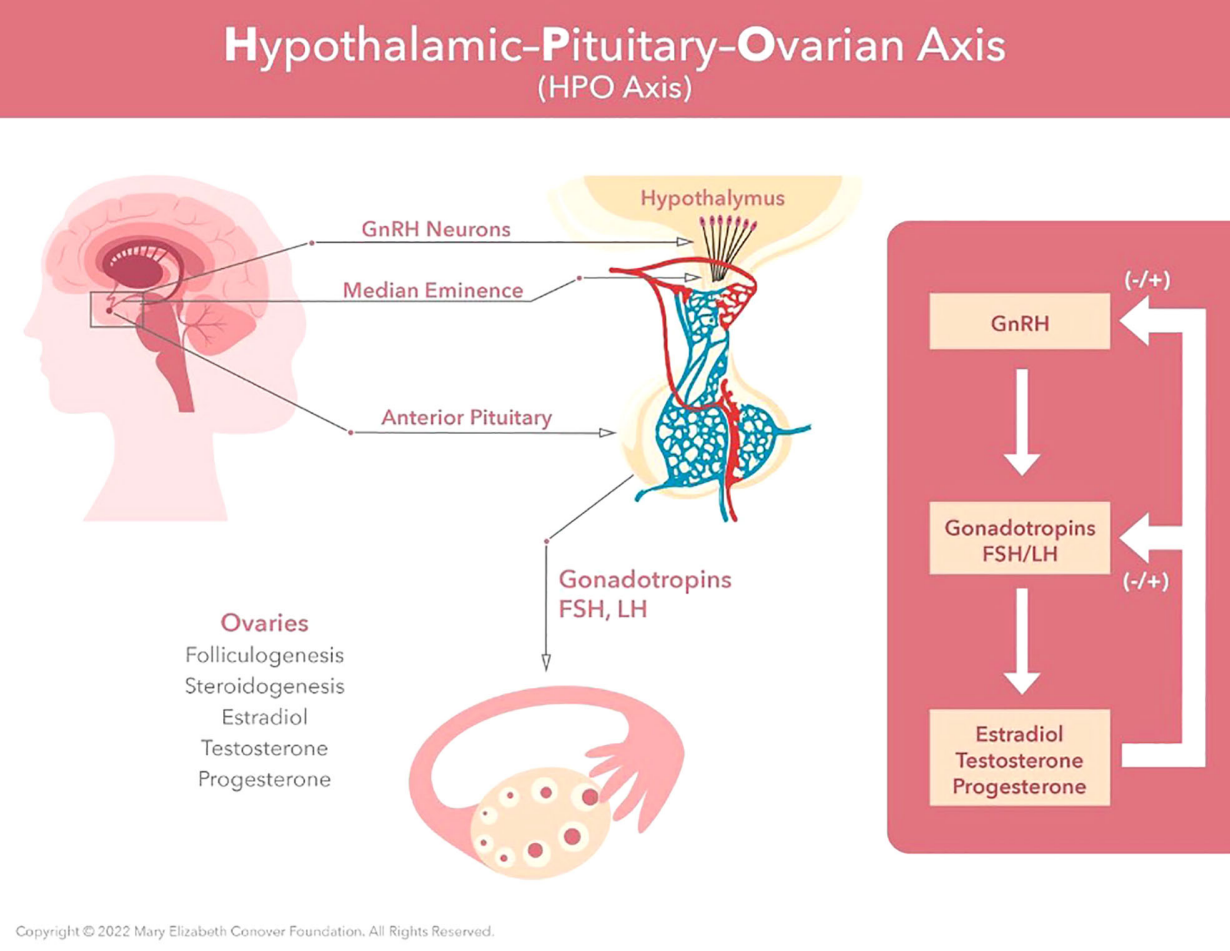
Menarche and the maintenance of normal regular menses requires normal function of the hypothalamic-pituitary-ovarian axis. GnRH neurons located in the hypothalamus produce regular pulses of GnRH at set intervals determined by estradiol and progesterone levels. These GnRH pulses are required for synthesis, storage, and release of FSH and LH by the anterior pituitary. Estradiol provides negative feedback to the central axis during follicular growth. This then switches to positive feedback under the influence of the preovulatory estradiol surge. This positive feedback induces the LH surge, which in turn induces ovulation.

A concerted action by what has been termed the hypothalamic-pituitary-ovarian (HPO) axis underlies menstrual cycle physiology. For a more detailed discussion of the menstrual cycle and its physiological control the reader is referred to Zeleznik and Plant (37). The production of estradiol and progesterone by the ovary during the menstrual cycle is dependent upon two protein hormones secreted by the pituitary gland and known as gonadotropins, follicle stimulating hormone (FSH) and luteinizing hormone (LH). The release of FSH and LH is, in turn, dependent on secretion of a hypothalamic releasing hormone now known as gonadotropin releasing hormone (GnRH) from neurons (nerve cells) located in a basal part of the brain termed the hypothalamus and situated immediately above the pituitary gland (**Figure 2**). Interestingly, the activity of hypothalamic GnRH neurons, which number several hundred, is synchronized to trigger brief episodes of GnRH secretion lasting a few minutes and occurring once every 1-8 h throughout the cycle. Such a pulsatile mode of the LH releasing hormone secretion was first proposed by Ernst Knobil before the isolation and characterization of GnRH was announced by Guillemin and Schally in 1971. Measuring LH in the circulation of female monkeys, Knobil’s laboratory observed distinct rhythmic episodes of LH secretion from the pituitary that occurred at approximately hourly intervals. The group proposed that this mode of gonadotropin secretion may be due to intermittent signals from the central nervous system (CNS) which, in turn, result in an increased production of LH releasing factor (ie GnRH) and the discharge of LH (38, 39). In 1978, the same laboratory demonstrated that pulsatile, but not continuous, GnRH stimulation of the pituitary was able to sustain LH and FSH secretion required for the menstrual cycle (40). These observations seeded the now well accepted idea that the intermittent signals from the CNS that lead to gonadotropin secretion originate from a neural timing mechanism known as the GnRH pulse generator. It is further known that the GnRH pulse generator is located within a distinct cluster of neurons referred to as the infundibular nucleus (aka arcuate nucleus) and transmits the intermittent CNS signal to the network of GnRH neurons via the neuropeptide transmitter, kisspeptin (41, 42).

The ovaries of young adult women typically contain a large, but ite, number of oocytes (eggs), each of which is individually encased in a spherical structure known as a primordial follicle. During the follicular phase of the menstrual cycle LH and FSH secretion, driven by the GnRH pulse generator, results in folliculogenesis: the growth of a cohort of primordial follicles, one of which will develop during the follicular phase into a fully mature follicle, the Graafian follicle, that will release its oocyte at the end of the follicular phase during the process of ovulation. The process of folliculogenesis culminating in the maturation of a single Graafian follicle is auto-regulated by circulating ovarian estradiol which restrains LH and FSH secretion. Such a control or servo system is known as a negative feedback loop – in this case estradiol provides the negative feedback signal. As the Graafian follicle matures it produces ever increasing amounts of estradiol, which, in addition to causing growth of the endometrium, serves as a hormonal signal for ovulation. This estrogen signal is relayed to the Graafian follicle indirectly via the pituitary: when blood levels of estradiol reach a threshold concentration (several hundred pg/ml) that is maintained for 2-3 days at the end of the follicular phase, the negative feedback action of low estradiol levels earlier in the follicular phase is replaced by a stimulatory or so called positive feedback action of estradiol that elicits a massive discharge of LH and FSH secretion (the preovulatory gonadotropin surge) that acts on the ovary to trigger ovulation. Following ovulation, the ruptured Graafian follicle is transformed into the corpus luteum, the structure responsible for the secretion of progesterone during the luteal phase of the menstrual cycle. The secretion of progesterone by the corpus luteum is also dependent on pulsatile LH secretion which together with that of FSH is driven by the GnRH pulse generator. With the decline in circulating estradiol levels that follows ovulation, a negative feedback control of gonadotropin secretion is reinstated with progesterone contributing to the negative feedback signal. Negative feedback actions of estradiol and progesterone are considered to occur at the level of both the hypothalamic pulse generator and directly at the pituitary. In the absence of fertilization and/or implantation (pregnancy), the corpus luteum regresses during the last week of the luteal phase resulting in menstruation and allowing for the initiation of the next cycle (see above).

The positive feedback action of estradiol is exerted directly at the pituitary and the role of the GnRH pulse generator in eliciting the preovulatory gonadotropin surge in the human female remains unclear. Nevertheless, it should not be forgotten that the rise in estradiol secretion from the maturing Graafian follicle that generates positive feedback and times the preovulatory gonadotropin surge is absolutely dependent on LH and FSH secretion driven by the GnRH pulse generator.

Very few primordial follicles mature into Graafian follicles; the vast majority begin to grow and develop but then undergo a process of degeneration and cell death known as atresia. Extensive atresia of developing follicles results in a progressive and marked decline in their numbers and typically during the 5-6^th^ decade of life the reserves of primary follicles are depleted to a level that is unable to maintain regular menstrual cyclicity. This results in an overall decline in circulating levels of estradiol and progesterone and represents the beginning of the menopausal transition. At this time, the reduction in ovarian steroid production weakens the negative feedback signal governing LH and FSH secretion leading to an elevation in circulating levels of these pituitary hormones, and in particular to those of FSH.

Perhaps surprisingly, the hypothalamic GnRH pulse generator is operational during fetal development and during infancy and at these stages of the life cycle the pituitary responds, as it does in adulthood, with increased LH and FSH secretion. The ovary of the fetus and infant, however, is not fully responsive to stimulation by the gonadotropins and therefore ovarian cyclicity does not occur at these early stages of development. Although the ovary becomes competent to respond fully to LH and FSH during childhood, by this age the GnRH pulse generator has been suppressed and the ensuing low levels of gonadotropin of childhood and juvenile development are insufficient to initiate menstrual cyclicity. The juvenile phase of development is terminated when the GnRH pulse generator is reactivated and puberty is initiated (43). This postnatal “on-off-on” pattern of GnRH pulse generator activity is currently conceptualized to result from the application of a neurobiological brake (also referred to as central restraint) during infancy and removal of this brake at the end of juvenile development. Neither, the nature of the neurobiological brake on the GnRH pulse generator during the greater part of prepubertal development nor the identity of the switches that time its application and removal is understood. Thus puberty, which is triggered by a re-activation or re-awakening of the GnRH pulse generator, remains a fundamental mystery of human biology. A premature re-awakening of the GnRH pulse generator which occurs with increasing frequency as girls approach the age of normal puberty (34 in 10,000 Danish girls at 8 years of age) will lead to precocious puberty and early menarche if left untreated (44).

In the context of the present review it is important to note that the GnRH pulse generator also serves as the CNS locus that integrates the influence of many environmental and pathophysiological factors on the menstrual cycle and therefore menstruation. For example, chronic undernutrition, high levels of energy expenditure, or stress will lead to disruption of the GnRH pulse generator, which results, sequentially, in 1) alterations in the pulsatile pattern of GnRH release, 2) decrease in gonadotropin secretion from the pituitary (hypogonadotropism), 3) reduction in release of ovarian estradiol and progesterone, 4) absence of cyclic growth of the endometrium, and 5) amenorrhea, i.e. the cessation of menstruation (45).

## The menstrual cycle as a vital sign

The menstrual cycle is indeed a vital sign of general health (46). It provides a powerful tool to assess normal development and detect the presence of a myriad of pathological conditions (47, 48). In a portion of cases, the onset of amenorrhea will be the first sign of serious pathology (49). Importantly, amenorrhea may indicate the presence of estradiol deficiency, a risk factor for osteoporosis, cardiovascular disease, cognitive decline, and even shortened life expectancy (50). Women who experience a delay in diagnosis of secondary amenorrhea are significantly more likely to have severely reduced bone mineral density (51). In girls or women of reproductive age, the presence of normal regular and predictable menstrual cycles (intervals of 21 to 35 days) provides evidence of normal development and ovarian function. Regular, predictable menses means the hypothalamus, pituitary, ovaries, uterus, and vagina are functioning normally regarding the endocrine function and the outflow tract of the axis.

The menstrual cycle with all its complexities requires that other endocrine systems and general metabolic pathways are intact. When these are disrupted by factors such as chronic stress, extreme exercise, eating disorders, and obesity the complex system governing the menstrual cycle is perturbed. Furthermore, genetic influences such as the fragile X premutation, X chromosome abnormalities, and galactose-1-phosphate uridyltransferase (GALT) point mutations (galactosemia) also contribute to perturbations of the menstrual cycle.

During the teen years, there is a tendency among parents and clinicians to view infrequent menses as a normal occurrence (26). In fact, the 95th percentile for the time interval between cycles is 90 days during the teen years. Thus, it is abnormal for an adolescent to be amenorrheic for greater than three months, even in the early gynecologic years (52). Identifying abnormal menstrual patterns throughout adolescence may permit early identification of potential health concerns for later in adulthood. A thorough evaluation of adolescent menstrual cycle disorders provides a window of opportunity for early diagnosis and treatment of conditions affecting the HPO axis. Both patients and clinicians need to view the ovary as a vital endocrine organ that helps maintain health, especially bone health and cardiovascular health.

## Amenorrhea

The literal meaning of amenorrhea is the absence of menses in a girl or woman of reproductive age. Amenorrhea is a normal physiologic state during pregnancy, lactation, and menopause. Chronic disease sometimes induces amenorrhea (45). In this situation, the underlying disorder will be diagnosed by presenting signs and symptoms specific to the disorder (53, 54). Amenorrhea is a common abnormality of the reproductive years. Amenorrhea outside of the physiologic states mentioned above indicates abnormal function or defect somewhere in the hypothalamic-pituitary-ovarian-uterine-vaginal axis. A girl or woman who presents with concerns about the absence of menses requires evaluation and management of the condition.

By age 15, 95% of girls will have had menarche, their first menses (55). Any adolescent who has not had menarche by age 15 deserves an evaluation for amenorrhea (46). The absence of other signs of pubertal development (e.g., breast and pubic hair development) by 13 years of age should also prompt investigation for delayed puberty.

## Primary and secondary amenorrhea overview

The prevalence of secondary amenorrhea not due to pregnancy, lactation, or menopause is approximately 3% to 4% (56, 57). As compared to primary amenorrhea, secondary amenorrhea has a shorter differential diagnosis, and the evaluation is much simpler. For example, menarche rules out complete androgen insensitivity syndrome (46,XY with female phenotype) and agenesis of the uterus or vagina (58). The four most common causes of primary amenorrhea are POI (48.5%), congenital absence of the uterus and vagina (16.2%), GnRH deficiency related to Kallman syndrome or other mechanism (8.3%), and constitutional delay of puberty (6.0%) (59, 60).

**Figure 3:**
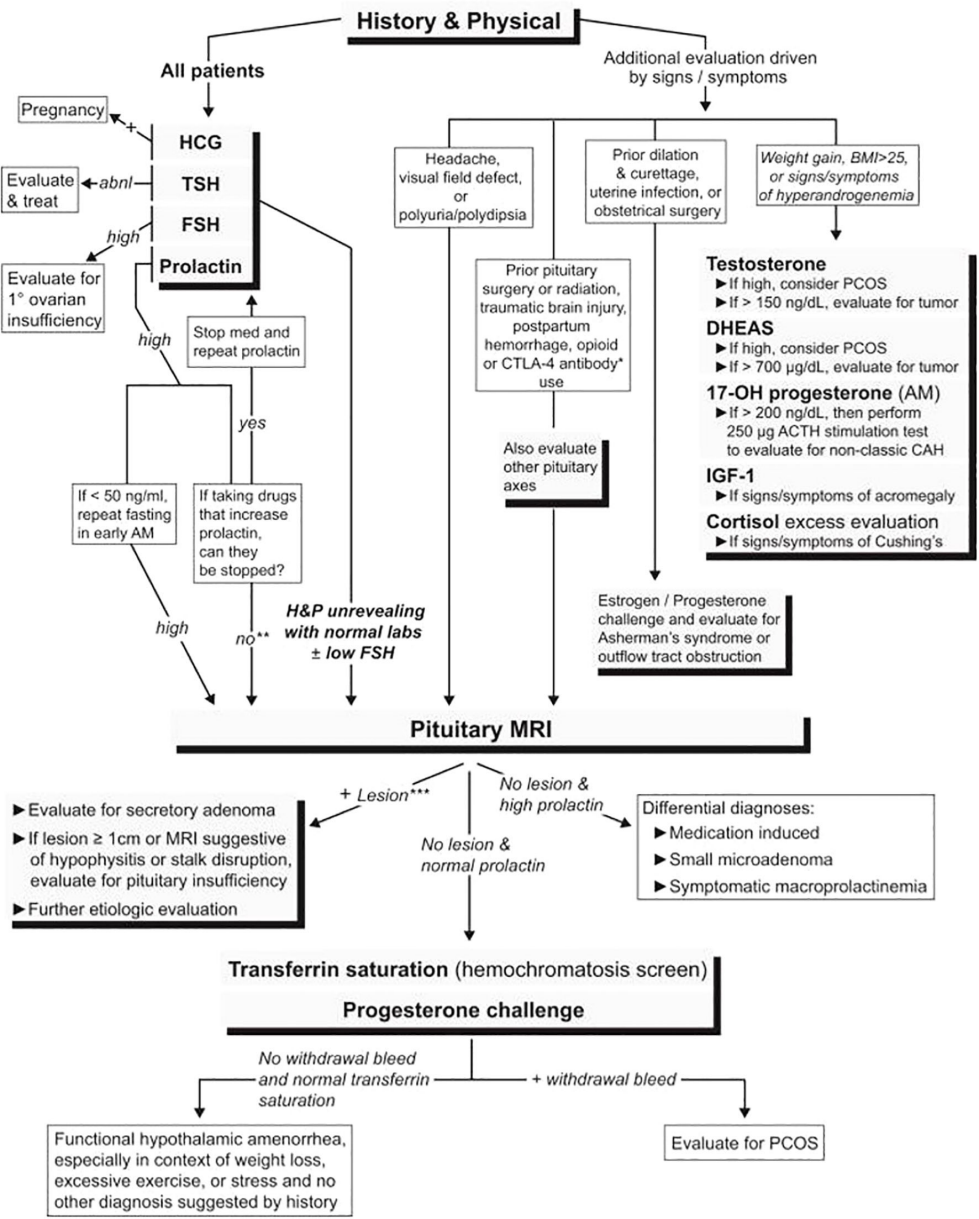
An algorithm for evaluating patients with secondary amenorrhea. abnl, abnormal; BMI, body mass index; DHEAS, dehydroepiandrosterone sulfate; PCOS, polycystic ovary syndrome; 17-OH progesterone, 17-hydroxyprogesterone; H&P, history and physical (61).

In evaluating secondary amenorrhea, the acute symptoms to be assessed on history are the presence of hot flashes, night sweats, vaginal dryness, insomnia, male pattern hair growth or balding, galactorrhea, headaches, visual disturbances, excess stress, and excess exercise, and/or excess dieting and changes in weight. However, POI may not always be associated with estradiol deficiency symptoms. The critical initial laboratory tests are an HCG pregnancy test (serum or urine), serum FSH, serum prolactin, TSH, testosterone, and a pelvic ultrasound. (**Figure 3**; **Table 1**). This evaluation will identify the most common causes of secondary amenorrhea (**Table 2**) (62). If there are signs and symptoms of androgen excess, measure serum testosterone levels (free or total to include fraction bound to plasma binding proteins). Amenorrhea may occur with sexual ambiguity or virilization, but usually, in these cases, amenorrhea is not the patient’s major complaint. Sexual ambiguity or virilization need to be evaluated as separate disorders, keeping in mind that amenorrhea is a component (63).

**Table 1:** Critical components: initial evaluation of secondary amenorrhea.

1. History and Physical Examination	
2. Rule out Pregnancy
3. Measure Serum FSH, TSH, Prolactin, Testosterone
4. Pelvic Ultrasound

**Table 2:** Causes of secondary amenorrhea (62).

Category	Approximate Frequency (Percent)
**High FSH** Primary Ovarian Insufficiency Normal Karyotype Abnormal Karyotype	12
**Low or Normal FSH** Secondary Ovarian Insufficiency Anorexia nervosa Undefined Hypothalamic Pituitary Cushing Syndrome	66
Chronic Anovulation including Polycystic Ovary Syndrome	
**High Prolactin**	13
**Uterine** (Asherman Syndrome)	7
**Hyperandrogenic State** Androgen Secreting Ovarian Tumor Congenital Adrenal Hyperplasia	2

## Two pathologic mechanisms of amenorrhea: central versus peripheral

Identifying the location of the lesion responsible for amenorrhea is critical to proper management. Is the problem centrally located, i.e., is the cause of amenorrhea in the hypothalamus or pituitary? (52) Or is the problem peripherally located, i.e., the cause of amenorrhea is dysfunction in the ovary, uterus, or outflow tract? (28).

Endocrinologists generally classify disorders of endocrine glands as “primary” or “secondary”. In the case of the ovary, *primary dysfunction (ovarian insufficiency or hypogonadism in women) is the result of a defect within the ovary itself that renders the gland unresponsive to gonadotropin stimulation. The central component of the axis (hypothalamus and pituitary) on the other hand is normal.* Hence, in primary hypogonadism serum gonadotropin levels are high. This is because in the absence of ovarian function estradiol and progesterone are not secreted and therefore feedback inhibition by these steroids on LH and FSH secretion at the central level (hypothalamus and pituitary) is lost. – Fuller Albright used the term Primary Ovarian Insufficiency when he first reported the condition, indicating the site of lesion in the HPO axis is in the ovaries. He concluded that the defect in function in these patients was primarily in the ovaries when he found evidence of high urinary FSH levels in these women (64).

Secondary hypogonadism in women with associated amenorrhea is the result of inadequate gonadotropin stimulation of the ovary and is therefore a central problem: if gonadotropin stimulation is provided in this condition the ovary will respond with menstrual cycles and menstruation.

Based on the discussion above, the term “Primary Ovarian Insufficiency” or POI conveys clearly the pathogenesis by identifying the lesion as being present in the ovaries. In contrast, the term “Premature Ovarian Insufficiency” is scientifically less exact as this condition may occur by either a primary ovarian cause or a secondary cause (hypothalamus or pituitary).

Finally, it is important to note that the term “primary amenorrhea” refers to a failure of menarche to ever occur spontaneously, and not necessarily the site of the underlying pathology. Failure of menarche to occur by 15 years of age is considered as primary amenorrhea.

## Primary ovarian insufficiency

POI affects up to 4% of women by age 40 and is rare in women less than 30. The condition is most commonly due to follicle dysfunction rather than follicle depletion (28, 65, 66). The diagnostic criteria are 1) women younger than 40 years, 2) four months of oligo-amenorrhea, and 3) two serum FSH levels in the menopausal range obtained at least one month apart (28). The disorder may become apparent from adolescence to age 40 years, with a mean age of onset of menstrual irregularity of 25 years (51).

Vasomotor symptoms and vaginal dryness are common in POI, yet their absence does not exclude the diagnosis (28, 67). Most cases are idiopathic, thought to be secondary to underlying genetic mutations, where >80 have been identified in a research capacity (68). However, irradiation, chemotherapy, tumors, autoimmune processes, and chromosomal irregularities can also cause POI (28). A karyotype should be obtained on all patients with a diagnosis of POI to identify Turner syndrome (or variants) (69). Patients diagnosed with POI merit testing for the *FMR1* gene premutation, which confers the risk of fragile X syndrome in their children (70, 71). Testing for 21 hydroxylase antibodies at initial diagnosis detects the presence of steroidogenic cell autoimmunity as the mechanism of POI and identifies the associated risk of potentially fatal autoimmune adrenal insufficiency (72).

Hormone replacement therapy (HRT) reduces associated vasomotor symptoms, sleep disorder, bone mineral density loss, and possibly cardiovascular risk. HRT should continue until the age of natural menopause (50 to 51 years) (73–79). Prospective, randomized, double blind, controlled trials provide the best evidence for clinical decision making (80). To date, there is only one published prospective, double blind, controlled clinical trial on the subject of HRT in POI, conducted by the USA National Institutes of Health Intramural Research Program (79, 81). The controlled trial, conducted over three years, supports using the NIH regimen referred to as physiologic HRT (P-HRT). The NIH P-HRT regimen provides 100 mcg of daily transdermal estradiol and 10 mg of oral medroxyprogesterone acetate daily for the first 12 days each calendar month (76). Clearly, transdermal estradiol is a superior choice to oral estrogens due to the well-documented reduced risk of potentially fatal venous thromboembolism by avoiding the adverse first pass effects on hepatic metabolism (82, 83). Other reported regimens provide 100-150 mcg of daily transdermal estradiol combined with a cyclical progestogen if the uterus is intact (for example, 10 mg oral medroxyprogesterone acetate or 200 mg micronized progesterone for 12-14 days per month) (79). Some women are willing to accept the increased risk of potentially fatal thromboembolism and elect to take oral HRT. In this case at least 2 mg of oral estradiol (with a progestogen if combined HRT is needed) or continuous use of the combined 30 mcg ethinyl estradiol oral contraceptive pill (OCP) have been shown in some studies to maintain bone density (84) and are potential alternatives, although not based on the best evidence, i.e., prospective, double blind, randomized controlled study. Shared decision making is important for HRT adherence and some women may opt for the “peer-friendly” yet more risky choice of the continuous OCP. The role of testosterone therapy in POI is unclear (76).

Approximately 5% to 10% of women who have POI conceive and deliver a healthy child (85). P-HRT does not suppress ovulation; therefore, barrier or IUD contraceptives are indicated in women who want to avoid pregnancy (28). The levonorgestrel intrauterine system can be combined with estradiol (transdermal preferably, or oral if necessary) as an HRT option which provides contraception. For optimal bone health, adolescent girls or women should avoid smoking, take 1200 mg of elemental calcium daily supplementation if dietary intake is insufficient, 800 IU of vitamin D daily, and regular weight-bearing exercises (28). Cardiac risk factors should also be assessed and managed appropriately (86). POI is associated with an increased risk of anxiety and depression and treatment including referral for psychological support may be needed (28, 86). A POI diagnosis introduces long-term challenges for patients and families. Clinicians who care appropriately offer ample time, sensitivity, and emotional support to the patient (87).

## Secondary ovarian insufficiency

### Functional hypothalamic amenorrhea

Functional hypothalamic amenorrhea is a cause of anovulation and estradiol deficiency. The mechanism may be suppression of the hypothalamic-pituitary axis from excessive exercise, loss of body weight, or stress (45, 52, 54, 88–90). The pathology is similar to the female athlete triad; both have associated oligo-amenorrhea, low energy availability, and decreased BMD (45, 54, 90, 91). Although functional hypothalamic amenorrhea is a diagnosis of exclusion, the evaluation typically demonstrates low or low-normal serum LH and FSH levels and low serum estradiol (45). Hypogonadism is an indication for bone mineral density evaluation (45, 54, 91). Treatment is aimed at correcting the underlying cause so as to restore ovulatory function. This may involve behavioral change, correcting nutritional deficiencies (e.g., increasing caloric intake), stress reduction, and gaining weight (45, 90, 91). A multidisciplinary team, including a clinician, nutritionist, registered dietician, and therapist, permits optimal management (45, 54, 91). Patients with severe bradycardia, hypotension, orthostasis, or electrolyte abnormalities as part of a restrictive eating disorder require inpatient treatment (45, 54). HRT as used in POI (described above) may be useful for managing estradiol deficiency.

Hormonal contraceptives may mask underlying pathology and are not a substitute for proper evaluation regarding the mechanism of amenorrhea. However, in cases where other contraceptive methods are unacceptable hormonal contraception may be indicated for patients at risk of pregnancy. Importantly, with remission of amenorrhea, ovulation may return prior to the return of menstruation. The Endocrine Society recommends against bisphosphonate use in this population as a means to improve bone mineral density (45, 54).

### Hyperprolactinemia

Elevated serum prolactin is a marker of disordered hypothalamic-pituitary function and is associated with amenorrhea due to central mechanisms (suppression of GnRH pulse generator). Common causes of hyperprolactinemia include pregnancy, a pituitary adenoma, and medications such as antipsychotics (92, 93). All patients with an elevated serum prolactin will require an MRI of the pituitary unless prolactin levels normalize after discontinuing inciting medications (93). In some cases, the risk of withdrawing medications such as antipsychotics may exceed the benefits of restoring ovarian function and this should only be done in coordination with the provider prescribing the medication(s) (93). Symptomatic prolactinomas may be treated with dopamine agonists or surgical resection (92, 93).

### Other causes

HPO axis disruption due to inflammation, ischemia, infiltration, infection, immunotherapeutic agents (ie CTLA4 inhibitors), rare disorders including GH-secreting pituitary tumors (acromegaly) or hypercortisolemia (Cushing’s syndrome) or trauma are other causes of amenorrhea (61).

## Other endocrine causes of secondary amenorrhea

### Polycystic ovary syndrome

PCOS presents with ovulatory dysfunction, biochemical or clinical androgen excess, and multicystic ovaries (94–97). The Rotterdam Consensus Criteria requires two of the features mentioned above for diagnosis; the Androgen Excess Society requires hyperandrogenism and one additional feature (94–96). Pelvic ultrasonography is not required for diagnosis (96, 97). Diagnostic accuracy in adolescence is challenging because anovulation and polycystic ovarian morphology can be transiently physiologic; therefore, hyperandrogenism and persistent oligomenorrhea are crucial to diagnosis (95, 96). Benefits of early management may outweigh the risks of delay for diagnostic certainty (95, 96). Markedly elevated serum androgens may indicate other conditions such as an adrenal or ovarian neoplasm (94, 96). PCOS can be associated with metabolic syndrome and insulin resistance. Patients should be screened for hypertension and a high body mass index at each visit and screened for dyslipidemia and impaired glucose tolerance at diagnosis and every three to five years (94, 96, 97). Recent evidence suggests simply demonstrating more than 23 follicles of any size in either ovary is a powerful tool by which to make the diagnosis of PCOS. This finding can not only accurately diagnose PCOS and is associated with metabolic–endocrine processes such as hyperandrogenism and insulin resistance.

It is proper to recommend healthy eating habits and regular exercise for all patients with PCOS. Weight loss in those patients with an elevated body mass index may restore regular menses and improve metabolic comorbidities (94, 96, 97). Combined hormonal contraceptives are first-line therapy for PCOS-related menstrual abnormalities, hirsutism, acne, and protection from endometrial cancer caused by unopposed estrogen secretion (94, 96, 97). Metformin is appropriate for patients with impaired glucose tolerance when lifestyle modification is unsuccessful or for those with contraindications to applicable contraceptives (96). Metformin is not effective for treating acne or hirsutism (96, 98). Letrozole (Femara), an antiestrogen, is a first-line ovulation induction therapeutic option for patients with PCOS and infertility. This agent is associated with higher ovulation, pregnancy, and live birth rates than clomiphene (94, 96, 97).

### Thyroid and adrenal disease

Both hypo- and hyperthyroidism can cause amenorrhea (45, 54, 60). Late-onset congenital adrenal hyperplasia (e.g., 21-hydroxylase deficiency) is a relatively common cause of hyperandrogenic amenorrhea and should be excluded prior to making the diagnosis of PCOS; an adrenocorticotropic hormone stimulation test can confirm the diagnosis if a serum 17-hydroxyprogesterone level is high (94, 96, 99). Adrenal or ovarian androgen-secreting tumors are rare but may be present in patients with rapid-onset virilization or markedly elevated serum androgens (73, 94, 96). If physical stigmata of cortisol excess are present such as moon facies, striae, or centripetal obesity, Cushing syndrome may be screened for with a 24-hour urinary free cortisol, late-night salivary cortisol, or dexamethasone suppression test (45, 54, 94, 96).

## Asherman syndrome

Amenorrhea related to intrauterine adhesions is known as Asherman syndrome (100). Intrauterine adhesions can result from lesioning of the basal layer of the endometrium caused by curettage of the recently pregnant uterus, especially if there is concurrent infection. The syndrome may also occur after hysteroscopic surgery, uterine artery embolization, or uterine infection, including tuberculosis. Contrast sonohysterography and hysterosalpingography are helpful in making this diagnosis if there is a failure to respond to an estrogen progestin withdrawal trial. Hysteroscopy confirms the final diagnosis (100).

## Proof of concept: a global digital hub for secondary amenorrhea

A fully integrated global healthcare hub based in the cloud would encompass a wide range of services and resources related to healthcare. This would include access to medical records, remote consultations with healthcare professionals, virtual health education programs, appointment scheduling, and more. By leveraging the power of cloud computing, such a hub could provide patients and healthcare providers with greater flexibility, convenience, and efficiency in accessing and delivering healthcare services.

The Mary Elizabeth Conover Foundation has addressed women's specific global educational needs specifically for women with POI, which most commonly presents as secondary amenorrhea. The condition creates diverse clinical needs. Thus, most individual clinicians lack the requisite experience, knowledge, and skill to evaluate and manage the entire needs of women with POI. The condition requires an integrated personal care approach with a team addressing all the clinical needs. The Mary Elizabeth Conover Foundation has been employing design thinking methodology to develop a model system to support a global approach to evaluating and managing POI (101). This approach is patient-centered and encourages empathy, creativity, and collaboration. By focusing on the patient and their needs, design thinking can lead to more innovative and effective solutions. The initiative at this point is specific to POI with a longer term goal of expanding global digital resources for any adolescent or woman experiencing amenorrhea.

Many women with POI perceive insufficient access to information, and/or dissatisfaction with the information provided (2). To explore the feasibility of creating a digital medical hub for these women via a professionally managed social media platform, the Conover Foundation has been managing a closed Facebook group which has grown to over 3,000 members (1). The purpose of the page is purely educational. The page is curated and administered by an experienced physician-scientist (LMN), who maintains coherent discussion threads, posts peer-reviewed resources, and assures accurate medical evidence. The POI Facebook team included access to a health coach, a POI Clinical Navigator, patient administrators for the page, and a personalized approach via videoconference.

**Figure 4:**
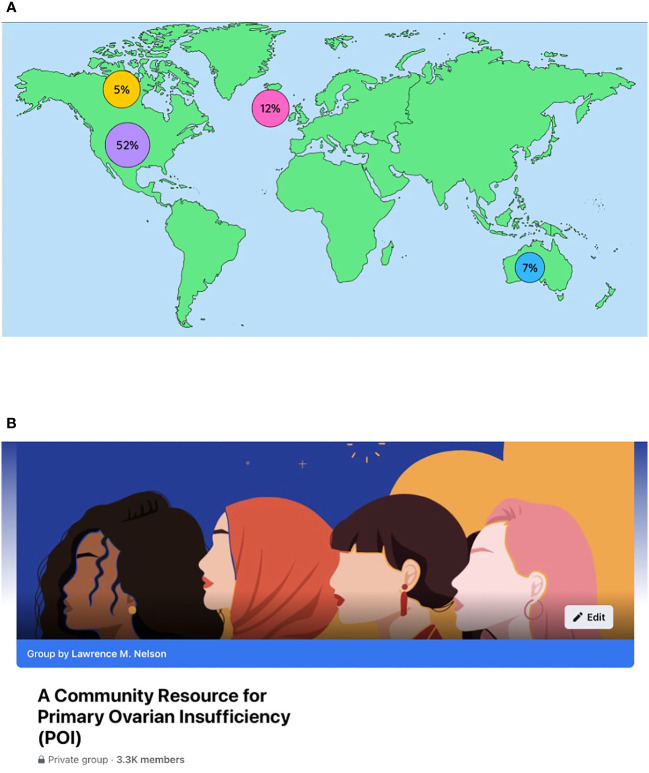
**(A)** A world map showing the countries with members representing more than 1% of the group. There were 100 different countries represented in total. **(B)** Homepage of the closed Facebook group “A Community Resource for Primary Ovarian Insufficiency ((POI)”.

In the year ending September 30, 2021, the number of members increased from 1084 to 2239, an increase of 107%. Members were from 100 different countries; 52% were from the United States, 12% United Kingdom, 7% Australia, 5% Canada, and 1% India. There were 896 posts and 8892 comments during the year. Cities with significant cohorts of membership (>20) include New York (39), London, UK (40), Melbourne (42), and Sydney (26) (102) (**Figure 4**). The page makes clear to members the purpose is purely educational. The page stresses the importance of obtaining medical advice and care from a personal clinician familiar with their specific health situation.

In this online forum, women read stories about international women with the condition and express their personal experiences with POI. They share stories about being informed of the diagnosis, the symptoms they have experienced, describe long-term health effects, mention treatments, report experiences with health services and health practitioners. They also provide assessments regarding the impact POI has had on their personal relationships and life in general.

Advanced computational and analytical tools can be employed to build quantitative networks. These networks cross molecular, biological, functional, and social organization (103). As part of the *My 28 Days^®^* initiative the Conover Foundation can combine mathematical models with data about secondary amenorrhea (e.g., biomonitoring samples, ‘omics screening, *in vitro* cellular toxicology, and in silico structure-based predictions). This approach could quantify the effects of environmental exposures and genetic variations. This approach, in turn, will facilitate prevention, early diagnosis, and treatment strategies. Recent advances in computational power and high throughput screening can be combined to build models based on pathways. Such models link molecular initiating events to outcomes. Such network models have been developed and applied in reproductive and developmental biology and toxicology (104–108). Examples include:

1. The identification of environmental exposures linking disruption of embryonic vascular growth and developmental defects.
2. Modeling endocrine disruption via critical nuclear hormone receptor signaling.
3. Connecting aromatase inhibition to female reproductive dysfunction in wildlife.
4. Annotating molecular and gene networks related to human testicular dysgenesis syndrome.

Our case report above is a clear example of the need to close the gap between knowledge and action in women’s health. The woman in this case learned about the benefits of P-HRT via communication on Facebook. This led her to the Conover Foundation page and ultimately to a clinician willing to prescribe P-HRT. The medical hub provided information to the clinician by educating the patient.

## Future steps

How shall we govern the *My 28 Days^®^* initiative? A practical and stable alliance requires a robust governance structure (109, 110), and must carry the effort through challenges and difficult times. Models of indigenous government were functioning quite successfully in America when Europeans first arrived (111–117). These indigenous governance models had a significant influence on the framing of the US constitution (118–121). The *My 28 Days^®^* global campaign envisions a governance structure based on these same egalitarian principles, i.e., respect for diversity and representative democratic cooperation in the community’s interest as a whole.

How will the My 28 Days® initiative engage the community? (122) Today’s world is media-saturated. Most marketing strategies attempt to persuade the masses through constant presence, repetition, and interruption. A stronger approach is to focus on a particular community or “tribe” and build relevance and value for their unique needs (123). The better aim is to create value for the individual and the specific community. The higher aim is to present a sustained and rewarding presence to the community in need by eventually providing a global cloud-based digital network of knowledge and care providers. The platform would serve the educational needs of clinicians as well as patients. We see a need to establish a menstrual cycle tribe. The word tribe arises from the Latin Tribus, referring to the three original tribes of Rome, divisions of the Roman people (124).

How will the Conover Foundation integrate the requirements of the *My 28 Days^®^* campaign into a global health care hub? Integration involves making decisions that combine disparate and even opposing ideas (125). The method in its basic form makes space for completely new ideas and ways of thinking. The medical hub must share ways in purpose, values, and rules of engagement to integrate and continually innovate (126). This glue holds things together when times get tough. Integrating diversity could be a strategy for innovation (127). Culture trumps strategy, regardless of how brilliant the strategic plan—transforming an “expert-centered” system into a “patient-centered” system requires cultural change (114). The “expert-centered” culture has functioned fine in many domains. Conover Foundation is functioning with and within this “expert-centered” culture to evolve positive change; change within the right direction benefits all and, ultimately, will appeal to all. Today’s best-performing companies understand the requirement to establish harmony with the present culture (114).

These types of organizations:

1. Create harmony between strategy and culture.
2. Identify a limited number of critical behaviors in need of change.
3. Emphasize the strengths of the prevailing culture.
4. Identify “thought leaders” who can bring other employees along.
5. Measure and monitor cultural changes.

## Discussion

Secondary amenorrhea is associated with a constellation of disorders. This symptom may lead to broad and profound health implications for the patient and extended family members. In specific situations, there is significant morbidity due to: (1) reduced bone mineral density, 2) cardiovascular disease related to estradiol deficiency, and 3) depression and anxiety related to hormonal deficiencies and fertility. Accumulating evidence also supports an important role for estradiol in maintaining brain health (128–131). Thus, the delay in diagnosis and management experienced by the woman reported here may impair her health over the long term.

Educational health resources have been shown to raise women’s awareness, potentially improving self-management, health related behaviors, informed decision making and health outcomes (132). Discussions related to estradiol replacement have become quite difficult as this important women’s health hormone has become a villain in the public eye following publication of the highly criticized US National Institutes of Health (NIH) Women’s Health Initiative (WHI) studies on postmenopausal hormone therapy (133–138). The major flaw in the study was average age of women was 63 years, well beyond the age when women first experience signs and symptoms of estradiol deficiency, as a result of the NIH WHI study, estradiol has been powerfully linked to risk of breast cancer, a fright which overshadows benefits related to heart, bone, brain health, and even longer life expectancy (81, 133, 139, 140). The discussions regarding the risks of estradiol deficiency and the benefits of estradiol replacement in young women are further complicated through erroneous extrapolation of the NIH WHI findings to adolescent girls and young women with POI (141).

Mobile health (mHealth) has great potential to improve care. This employs modern telecommunications to deliver care remotely. This technology is transforming healthcare and biomedical research (142–146). mHealth increases access to health education and makes care more convenient. mHealth presents a mechanism to close the knowledge-action gap in secondary amenorrhea and other health disorders. mHealth also can integrate research and patient care and improve research efficiency. These capabilities accelerate innovation (109, 110, 147, 148). The patient is the customer in medical care and research. mHealth can engage the patient as a key collaborator. Engaging the customer is a fundamental concept in design thinking. Novel solutions to recalcitrant problems arise. mHealth keeps patients at the center and empowers them to improve their care. mHealth permits patients to share data and experiences about symptoms, treatments, and behaviors.

The strategy of the Conover Foundation is to focus on the needs, goals, and aspirations of girls and women with amenorrhea. This approach leverages affiliation, attracting community (tribal) members with shared traits (149). This suits the character and values of those involved and addresses desires for membership, self-expression, and identity. The membership provides a status marker to empower the individual. This provides a strong sense of belonging to a group (150). The result leads to creating ambassadors who spread positive word of mouth about the *My 28 Days^®^* initiative, the associated digital medical hub for care, and the research alliance. Tribal engagement delivers powerful messaging. The idea is to look constantly for fresh ways to maintain relevance and create more value for the community. Clinicians are also needed as ambassadors to afford their patients the opportunity to connect with other adolescents and women through the digital medical hub.

Establishing an innovative, collaborative system for secondary amenorrhea must encourage clear communication. This means ensuring claims about the community are authentic and foster solidarity. This means supporting reasonable action and appealing to intrinsic motivations (151). The Conover Foundation favors an approach that engages and embraces human generosity instead of assuming pure self-interest as usually the first driver. Western culture has for generations based community models on the belief that mortals are selfish at the core (152). As a result, communities tend to focus mainly on rewards, monetary incentives, and punishments.

Real-world examples of community projects like Wikipedia, open-source software, and Craigslist, provide evidence that people are more cooperative and behave far less selfishly than generally assumed (115). Systems established on cooperation instead of incentive are often more stable and prosperous. Evolution may favor those that collaborate and societies that include such individuals (153). When Lakota people pray together, saying, “*mitakuye Oyasin*,” translated “all my relations,” they embody and validate this community approach (154). In sum, integration means making space for others who are different in culture and domain of experience. Integration involves rhetoric, friendship, authority, emotional space, and race (155).

## Conclusion

The experience with the case report presented above proves the feasibility of creating a digital medical hub for secondary amenorrhea. Such an online educational platform combines the community-building power of social media with the power of peer-reviewed research. The goal is to grow an international digital medical home for women with POI. In addition, the resource guides evaluation and management based on evidence and is a focal point around which to consolidate the *My 28 Days^®^* menstrual cycle global network of education, advocacy, care, and research (30).

A presentation of secondary amenorrhea deserves prompt attention to avoid delay in diagnosis of a potentially serious condition. In most cases, the initial minimal laboratory evaluation of this symptom will be to test for pregnancy, and if negative, measure serum FSH, prolactin, TSH and testosterone, and obtain a pelvic ultrasound examination. An international digital medical hub dedicated to secondary amenorrhea would help close the knowledge to action gap that women with this condition face regularly. This international digital medical hub, ​​connecting patients, community health providers, and academia in real-time, would create a community of practice to advance education, advocacy, care, and research. Such an initiative takes full advantage of internet and mobile device communication systems. We refer to this global digital health initiative as *My 28 Days^®^* and have shown proof of concept in regard to POI (30). The term 28 Days^®^ is a registered trademark of the Mary Elizabeth Conover Foundation, all rights reserved (30).

## Data availability statement

Personal identifiers in this case report are confidential and not available. Requests to access the datasets should be directed to Doc@ConoverFoundation.org.

## Ethics statement

Written informed consent was obtained from the individual(s) for the publication of any potentially identifiable images or data included in this article.

## Author contributions

LN conceived the need for this report and composed the first draft. All the other coauthors contributed important intellectual content to improve the quality of the manuscript. All authors contributed to the article and approved the submitted version.
